# Impact of Elevated Lipoprotein A on Major Adverse Cardiovascular Events and the Role of Traditional Risk Factors in Patients Undergoing Percutaneous Coronary Intervention

**DOI:** 10.7759/cureus.77630

**Published:** 2025-01-18

**Authors:** Nisar Ahmad Zemawal, Salman Shah, Mustaqeem Shah, Mubashir Qadar Khattak, Imran Qadar Khattak, Talha Mazhar, Riffat Shaheen, Muhammed Irfan

**Affiliations:** 1 Medicine and Surgery, Lady Reading Hospital, Peshawar, PAK; 2 Internal Medicine, Hayatabad Medical Complex, Peshawar, PAK; 3 Internal Medicine, Lady Reading Hospital, Peshawar, PAK; 4 Medicine and Surgery, Saidu Medical College, Swat, PAK; 5 Medicine and Surgery, Divisional Headquarters Teaching Hospital, Mirpur, PAK; 6 General Medicine, Hayatabad Medical Complex, Peshawar, PAK

**Keywords:** cardiovascular risk, lipoprotein(a), mace, pci, traditional risk factors

## Abstract

Background: Elevated lipoprotein(a) (Lp(a)) is a recognized independent risk factor for adverse cardiovascular outcomes, particularly in patients undergoing percutaneous coronary intervention (PCI).

Objective: This study aims to evaluate the impact of elevated Lp(a) on major adverse cardiovascular events (MACE) in patients undergoing PCI and investigate the interplay and independent contributions of Lp(a) and traditional cardiovascular risk factors.

Materials and methods: A prospective cohort study was conducted over two years, including 360 patients undergoing PCI. Participants were divided into high and normal groups according to their baseline Lp(a) levels. To record MACE, which includes myocardial infarction, stroke, repeat revascularization, and cardiovascular mortality, clinical, demographic, and laboratory data were gathered, and patients were monitored for 24 months. The association between Lp(a) levels, MACE, and conventional risk variables was examined using Cox proportional hazards models.

Results: Those with normal Lp(a) (18.69 ± 5.39 mg/dL) had substantially lower rates of MACE, such as myocardial infarction (HR = 1.89, p = 0.002), stroke (HR = 1.48, p = 0.039), and cardiovascular mortality (HR = 2.23, p = 0.001), than those with raised Lp(a) (79.31 ± 35.13 mg/dL). The raised group had a considerably reduced event-free survival rate (74.13% vs. 91.76%, p < 0.001). The raised group was more likely to have traditional risk factors, including diabetes and hypertension, which increased the negative consequences.

Conclusion: Elevated Lp(a) significantly increases the risk of MACE in PCI patients, and this risk is further modulated by the presence of traditional cardiovascular risk factors, emphasizing the need for comprehensive risk stratification.

## Introduction

The majority of instances of cardiovascular diseases (CVD), which continue to be the world's leading cause of morbidity and death, are caused by coronary artery disease (CAD) [1,2]. Elevated lipoprotein(a) (Lp(a)) has become a crucial independent risk factor among the several variables causing the onset and progression of CAD [3]. Apolipoprotein(a) is a distinct component of Lp(a), a particle that resembles low-density lipoprotein (LDL) [4]. It has pro-thrombotic and pro-atherogenic qualities that may increase the risk of cardiovascular events [5]. An important biomarker in modern cardiovascular risk assessment, elevated Lp(a) levels have been repeatedly associated with an increased risk of myocardial infarction, stroke, and aortic stenosis [6]. Moreover, Lp(a) has a predictive impact that may rival or complement traditional cardiovascular risk factors, such as diabetes, hypertension, and dyslipidemia.

Multiple risk factors are often present in patients undergoing percutaneous coronary intervention (PCI), increasing the chance of unfavorable cardiovascular outcomes [7]. PCI does not remove the underlying risk of further cardiovascular events despite its effectiveness in reducing ischemia and restoring coronary blood flow [8]. Conventional risk factors that continue to have a significant role in post-PCI outcomes include hypertension, diabetes, hyperlipidemia, and smoking history. However, further research is necessary to fully understand how these variables interact with higher Lp(a) in this high-risk group [9].

Finding and reducing residual risk factors is still a top priority, even though improvements in pharmacological and interventional therapies have improved long-term survival for PCI patients [10]. Despite its well-established role in atherosclerosis, Lp(a) measurement is not always incorporated into standard clinical practice, and its impact on PCI patient outcomes is still undervalued [11]. Furthermore, little is known about how conventional cardiovascular risk factors influence the effect of high Lp(a) on the prognosis after PCI [12].

Our research fills this important gap by examining the connection between high Lp(a) levels and major adverse cardiovascular events (MACE) in patients undergoing PCI. It also assesses the impact of conventional risk variables on this connection to improve risk classification and individualized treatment plans.

Objective

This study aims to assess the impact of elevated Lp(a) levels on MACE in patients who underwent PCI and evaluate the modifying role of traditional cardiovascular risk factors on this relationship.

## Materials and methods

Study design and setting

The study was conducted at Hayatabad Medical Complex (HMC), a high-volume tertiary care hospital in Peshawar, Pakistan. HMC serves a diverse patient population, including individuals from urban and rural areas of Khyber Pakhtunkhwa Province and neighboring regions. Its status as a referral center for advanced cardiovascular care, including PCI, ensures access to a wide demographic and a high volume of cases, making it an ideal setting for studying cardiovascular risk factors. Patient recruitment occurred continuously throughout the two-year study period (January 2022 to December 2023) to ensure a comprehensive representation of the patient population undergoing PCI. The baseline Lp(a) levels were obtained within the first 24 hours of hospital admission to ensure clinical stability and reproducibility of results, as levels measured during acute events may be transiently altered.

Inclusion and exclusion criteria

Adult patients aged 18 years or older undergoing PCI at HMC between January 2022 and December 2023 were included in the study, provided they had baseline Lp(a) levels obtained within the first 24 hours of hospital admission and gave informed consent. The study encompassed patients with various cardiovascular conditions, including acute coronary syndrome and chronic coronary disease, to evaluate the impact of elevated Lp(a) across different stages of CAD. Lp(a) was measured using standard immunoassay methods in the HMC laboratory, with high Lp(a) levels defined as >50 mg/dL in accordance with clinical guidelines and prior research. Exclusion criteria included patients with prior coronary artery bypass grafting or PCI, terminal conditions or cancers with a life expectancy of less than one year, insufficient clinical data, or incomplete follow-up, as well as patients using lipid-lowering medications specifically targeting Lp(a), which could confound the results by artificially lowering Lp(a) levels. Diabetes and hypertension were diagnosed based on established criteria, including fasting blood glucose levels ≥126 mg/dL, HbA1c ≥6.5%, and blood pressure ≥140/90 mmHg on two separate occasions or documented use of relevant medications.

Sample size

The estimated prevalence of increased Lp(a) in PCI patients and its correlation with MACE were used to determine the sample size. The necessary sample size was determined to be around 323 individuals using a method for cohort studies, with an expected prevalence of 30% for high Lp(a) [13], a 95% confidence level (Z = 1.96), and a 5% margin of error. A 10% dropout rate was considered to account for any non-compliance and dropouts, resulting in an adjusted sample size of around 360 participants. As a result, sufficient statistical power was guaranteed to identify noteworthy variations and evaluate the interplay with conventional risk variables.

Data collection

Hospital records and patient interviews were used to collect baseline demographic, clinical, and laboratory data, including Lp(a) levels and key cardiovascular risk factors such as diabetes, hypertension, smoking, and dyslipidemia, as well as information related to PCI. To evaluate the incidence of MACE, including myocardial infarction, stroke, repeat revascularization, and cardiovascular mortality, patients were monitored at six, 12, 18, and 24 months post-PCI. Follow-up data were gathered through a combination of in-person clinic visits and phone interviews at these intervals. Each follow-up involved a thorough review of clinical records, patient-reported outcomes, and any additional evaluations deemed necessary to assess short-term and long-term results.

MACE was defined as myocardial infarction (characterized by elevated cardiac biomarkers with ischemic symptoms or ECG changes), stroke (a focal neurological deficit lasting >24 hours, confirmed by imaging), repeat revascularization, and cardiovascular mortality. These diagnoses were made in accordance with established clinical guidelines, and the manuscript provides references to these diagnostic criteria for clarity and consistency.

Statistical analysis

Statistical analyses were conducted using SPSS Statistics version 26.0 (IBM Corp. Released 2019. IBM SPSS Statistics for Windows, Version 26.0. Armonk, NY: IBM Corp.). Descriptive statistics were used to summarize baseline characteristics, with continuous variables expressed as means ± standard deviations and categorical variables as frequencies and percentages. The normality of continuous data was assessed using the Shapiro-Wilk test. For normally distributed variables, parametric tests, such as independent t-tests, were applied, whereas non-parametric tests, including the Wilcoxon-Breslow test, were used for non-normally distributed data. To evaluate the association between elevated Lp(a) levels and the occurrence of MACE, Cox proportional hazard models were employed, adjusting for conventional cardiovascular risk factors such as diabetes, hypertension, smoking, and dyslipidemia. Interaction effects between Lp(a) and other risk factors, such as diabetes and hypertension, were explored by incorporating interaction terms (Lp(a) × diabetes, Lp(a) × hypertension) into the models. A likelihood ratio test was used to compare models with and without the interaction terms. Missing data were excluded from the analysis, and no imputation was performed. The Wilcoxon-Breslow test compared event-free survival between groups, emphasizing early survival differences and complementing the Cox models by not assuming proportional hazards over time. P-values of less than 0.05 were considered statistically significant.

Measures to minimize potential biases

To minimize potential biases, several measures were implemented during the study design and execution. First, patient recruitment was conducted continuously over a two-year period to ensure a comprehensive representation of individuals undergoing PCI at the study center. Baseline Lp(a) levels were measured within 24 hours of hospital admission to avoid transient fluctuations during acute events. To ensure uniformity and accuracy, a standardized protocol was used for data collection, including laboratory testing, clinical assessments, and follow-up evaluations. Selection bias was mitigated by applying broad inclusion criteria that captured a diverse range of patients with acute and chronic CAD. While some exclusions were necessary for scientific rigor (e.g., terminal conditions, prior interventions, or insufficient follow-up), these criteria were predefined to maintain transparency. Loss to follow-up was minimized through scheduled in-person visits and phone interviews at six, 12, 18, and 24 months, with efforts made to re-contact participants who missed follow-ups. Additionally, statistical analyses were adjusted for conventional cardiovascular risk factors, such as age, gender, diabetes, hypertension, smoking, and dyslipidemia, to control for potential confounders. Together, these steps enhanced the validity and reliability of the study findings while reducing the impact of selection, measurement, and analytical biases.

Ethical considerations

The Institutional Review Board of Hayatabad Medical Complex examined and approved the research protocol (approval number: 26/OD/HMC/2022). All individuals provided written informed consent prior to their involvement in the research. No data revealing patient identity (e.g., names, addresses, or contact information) were collected to preserve patient confidentiality. Instead, patient numbers as identifiers were used to link patient records securely. Data access was restricted to authorized research personnel to ensure confidentiality.

## Results

The research had 360 people who were split evenly into two groups according to their increased or normal Lp(a) levels. The mean age of participants with raised Lp(a) was 60.29 ± 9.75 years, which was greater than the mean age of the normal Lp(a) group (56.52 ± 10.96 years). In contrast to the normal Lp(a) group, which consisted of 135 men and 45 females, the raised group contained more males (n = 145) than females (n = 35). The raised group had higher rates of smoking history (82 vs. 71), diabetes mellitus (70 vs. 58), hypertension (118 vs. 102), and dyslipidemia (93 vs. 81). Compared to the normal Lp(a) group (18.69 ± 5.39 mg/dL), the raised group's mean Lp(a) levels were substantially higher (79.31 ± 35.13 mg/dL). The raised group had somewhat greater LDL cholesterol (123.14 ± 36.52 vs. 118.64 ± 30.21 mg/dL) and total cholesterol (201.86 ± 46.13 vs. 195.42 ± 41.28 mg/dL) but lower high-density lipoprotein (HDL) cholesterol (47.25 ± 14.17 vs. 50.45 ± 12.36 mg/dL), according to other test results. Additionally, the raised group had greater triglyceride levels (168.54 ± 78.21 vs. 157.84 ± 68.15 mg/dL). Interestingly, the increased group had a history of prior myocardial infarction (60 vs. 55), more PCI stents (150 vs. 140), and somewhat worse left ventricular function (72 vs. 68 instances). P-values and test statistics were calculated using the independent t-test for continuous variables (e.g., age: t = 3.41, p = 0.001) and the chi-square test for categorical variables (e.g., gender: χ² = 1.52, p = 0.217), with p < 0.05 values indicating statistical significance; these results highlight meaningful differences in clinical and laboratory characteristics between the groups, warranting further exploration of their potential clinical implications. Table 1 shows the details.

**Table 1:** Baseline demographic, clinical, and laboratory data of participants t = independent t-test for continuous variables, χ² = chi-square test for categorical variables, p-values <0.05 indicate statistical significance, Lp(a): lipoprotein(a), SD: standard deviation, LDL: low-density lipoprotein, HDL: high-density lipoprotein, PCI: percutaneous coronary intervention, MI: myocardial infarction

Variable		Elevated Lp(a) (n = 180)	Normal Lp(a) (n = 180)	p-value	Test statistic
Age (years)	Mean ± SD	60.29 ± 9.75	56.52 ± 10.96	<0.001	t = 3.96
Gender (n; %)	Male	145 (80.56%)	135 (75.00%)	0.182	χ² = 1.79
	Female	35 (19.44%)	45 (25.00%)		
Clinical characteristics	Hypertension	118 (65.56%)	102 (56.67%)	0.08	χ² = 3.06
	Diabetes mellitus	70 (38.89%)	58 (32.22%)	0.185	χ² = 1.75
	Smoking history	82 (45.56%)	71 (39.44%)	0.249	χ² = 1.33
	Dyslipidemia	93 (51.67%)	81 (45.00%)	0.201	χ² = 1.63
Laboratory data	Lp(a) levels (mg/dL)	79.31 ± 35.13	18.69 ± 5.39	<0.001	t = 21.57
	LDL cholesterol (mg/dL)	123.14 ± 36.52	118.64 ± 30.21	0.173	t = 1.37
	HDL cholesterol (mg/dL)	47.25 ± 14.17	50.45 ± 12.36	0.042	t = -2.04
	Total cholesterol (mg/dL)	201.86 ± 46.13	195.42 ± 41.28	0.198	t = 1.29
	Triglycerides (mg/dL)	168.54 ± 78.21	157.84 ± 68.15	0.196	t = 1.29
PCI-related details	PCI type (stent/angioplasty)	150 (83.33%)	140 (77.78%)	0.187	χ² = 1.74
	Previous MI	60 (33.33%)	55 (30.56%)	0.601	χ² = 0.27
	Left ventricular function	72 (40.00%)	68 (37.78%)	0.693	χ² = 0.16

**Figure 1:**
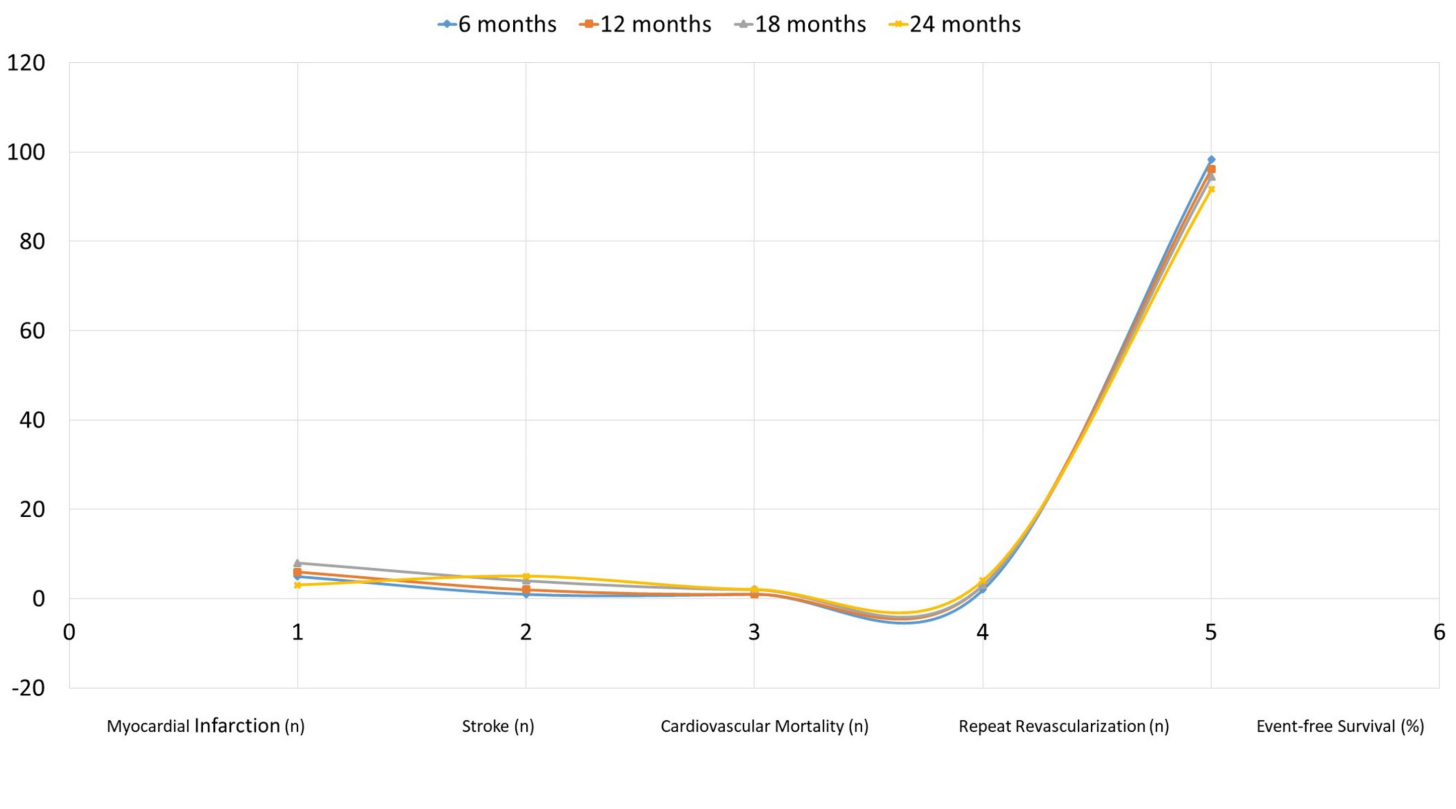
Incidence of MACE in the normal Lp(a) group (n = 180) MACE: major adverse cardiovascular events, Lp(a): lipoprotein(a)

The incidence of MACE during a 24-month period in the normal Lp(a) group (n = 180) is shown in Figure 1. At six months, there are five myocardial infarction instances; at 24 months, there are just three. At six months, there is just one stroke case; at 24 months, there are five. While cardiovascular mortality stays mostly constant, rising from one patient at six months to two at 24 months, repeat revascularization increases from two at six months to four at 24 months.

**Figure 2:**
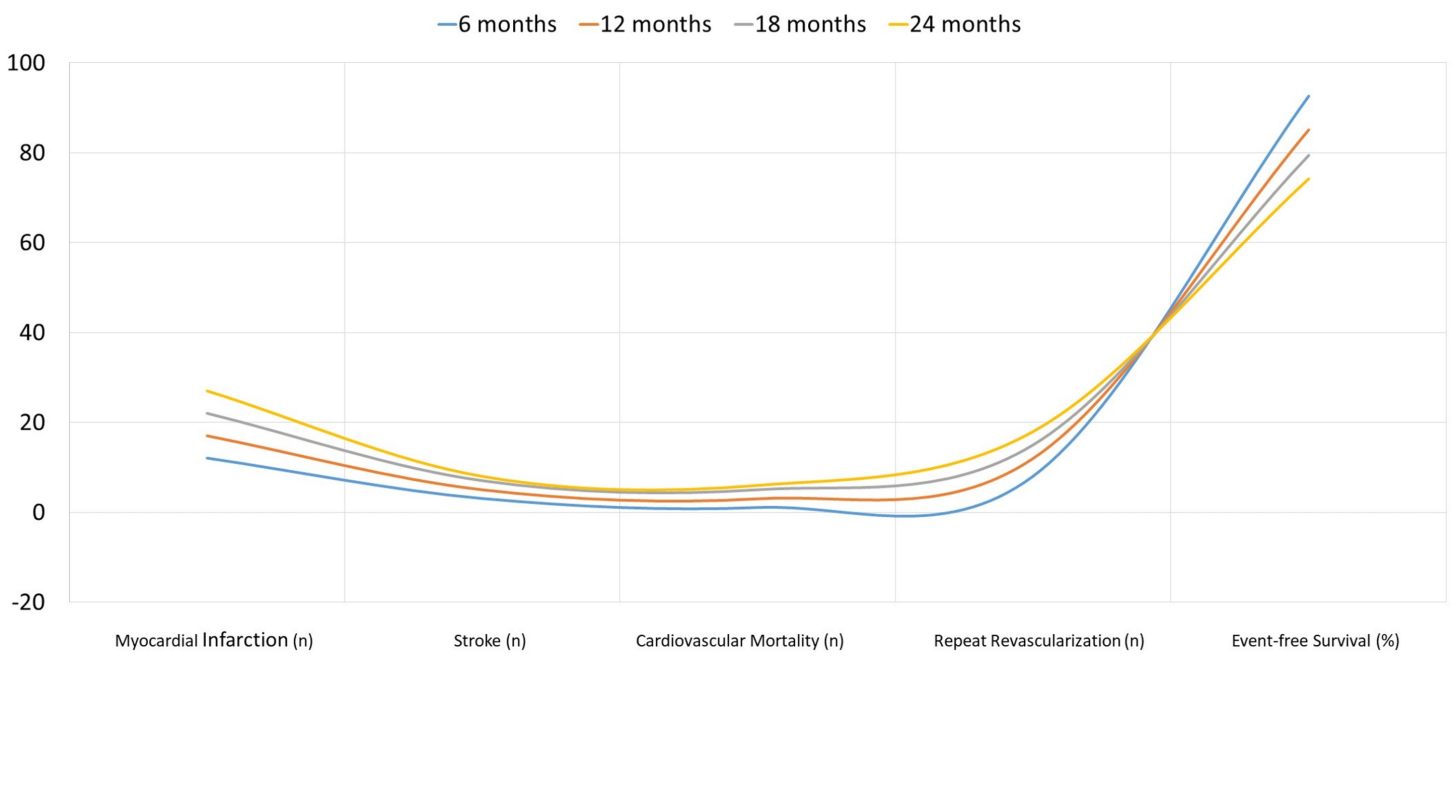
Incidence of MACE in the Lp(a) elevated group (n = 180) MACE: major adverse cardiovascular events, Lp(a): lipoprotein(a)

The incidence of MACE during a 24-month period is shown in Figure 2 for the Lp(a)-raised group (n = 180). By 24 months, there are 27 individuals who have had myocardial infarction, up from 12 at six months. Stroke cases increase from three at six months to eight at 24 months. Cardiovascular mortality increases from one patient at six months to six at 24 months, and repeat revascularization increases from eight at six months to 18 at 24 months.

Kaplan-Meier survival curves in Figures 1-2 for more precise visualization of MACE progression over 24 months. The survival curves show earlier divergence and sustained separation over time, emphasizing the long-term risk associated with raised Lp(a) levels. These curves, supported by the Wilcoxon-Breslow test (p < 0.001), highlight the lower event-free survival rates in the raised Lp(a) group compared to the normal Lp(a) group. Trends in MACE incidence, such as increased myocardial infarction and cardiovascular mortality over time, suggest significant clinical concerns for the raised group.

After controlling for conventional cardiovascular risk factors (age, gender, smoking, hypertension, diabetes mellitus, dyslipidemia, and prior myocardial infarction), Table 2 presents the outcomes of the Cox proportional hazards model for MACE in the elevated Lp(a) group (n = 180). The findings indicate that elevated Lp(a) significantly increases the risk of myocardial infarction (HR = 1.89, p = 0.002), stroke (HR = 1.48, p = 0.039), repeat revascularization (HR = 1.67, p = 0.017), and cardiovascular death (HR = 2.23, p = 0.001). These hazard ratios demonstrate that patients with elevated Lp(a) face a notably higher likelihood of adverse cardiovascular events, with cardiovascular death being particularly pronounced (HR = 2.23). All p-values are below 0.05, underscoring the clinical significance of elevated Lp(a) as an independent risk factor for MACE.

**Table 2:** Cox proportional hazard model for MACE in the elevated Lp(a) group (n = 180) adjusted for traditional cardiovascular risk factors MACE: major adverse cardiovascular events, Lp(a): lipoprotein(a)

Event type	HR	95% CI	p-value
Myocardial infarction	1.89	1.32-2.71	0.002
Stroke	1.48	1.02-2.14	0.039
Repeat revascularization	1.67	1.18-2.37	0.017
Cardiovascular death	2.23	1.45-3.42	0.001

The 24-month event-free survival rates for the normal and elevated Lp(a) groups are presented in Table 3. The elevated Lp(a) group demonstrates a significant decline in event-free survival, starting at 92.54% at six months and dropping to 74.13% at 24 months. In contrast, the normal Lp(a) group maintains higher survival rates, decreasing from 98.32% at six months to 91.76% at 24 months. The Wilcoxon-Breslow test revealed a statistically significant difference between the two groups (p < 0.001), indicating that higher Lp(a) levels are associated with a lower event-free survival rate over time. This highlights the potential clinical significance of Lp(a) as a risk factor in cardiovascular outcomes.

**Table 3:** Comparison of event-free survival between elevated and normal Lp(a) groups (Wilcoxon-Breslow test)

Group	6 months (%)	12 months (%)	18 months (%)	24 months (%)	p-value
Elevated Lp(a)	92.54	85.03	79.42	74.13	<0.001
Normal Lp(a)	98.32	96.16	94.41	91.76	

## Discussion

Our study demonstrates that elevated Lp(a) levels are a significant independent risk factor for MACE in patients undergoing PCI. Compared to the normal Lp(a) group, patients with elevated Lp(a) exhibited higher rates of myocardial infarction, stroke, repeat revascularization, and cardiovascular death, aligning with prior research highlighting Lp(a)’s pro-atherogenic and pro-thrombotic properties as key contributors to adverse cardiovascular outcomes. For instance, the HR of 1.89 (p = 0.002) for myocardial infarction in the elevated Lp(a) group is consistent with earlier findings that patients with high Lp(a) levels face nearly double the risk of myocardial infarction [13,14], emphasizing the clinical importance of this marker in predicting ischemic events.

The event-free survival rate at 24 months was significantly lower in the elevated Lp(a) group (74.13%) compared to the normal Lp(a) group (91.76%, p < 0.001). This finding is consistent with prior studies demonstrating the negative impact of high Lp(a) levels on long-term cardiovascular outcomes in CAD patients [15]. Additionally, the elevated Lp(a) group experienced an increase in stroke incidence (from three to eight cases between six and 24 months) with an HR of 1.48 (p = 0.039). These results align with earlier studies linking elevated Lp(a) levels to an increased stroke risk [16], reinforcing the need for heightened surveillance and intervention in high-risk individuals.

The interplay between elevated Lp(a) and conventional cardiovascular risk factors further underscores the complexity of risk stratification. The elevated Lp(a) group exhibited higher rates of hypertension (65.56% vs. 56.67%), diabetes, and dyslipidemia, which likely exacerbate adverse outcomes. Previous studies have shown that hypertension amplifies Lp(a)'s impact on atherosclerosis, a finding supported by our results [17]. This interaction suggests that the presence of these comorbidities should prompt closer monitoring and more aggressive management strategies in patients with high Lp(a).

Our findings also underscore the role of elevated Lp(a) in restenosis after PCI, as evidenced by the increased need for repeat revascularization in the elevated Lp(a) group (HR = 1.67, p = 0.017). Mechanistically, Lp(a) is known to promote smooth muscle cell proliferation and vascular inflammation, processes that are critical to restenosis [18]. Additionally, elevated levels of triglycerides, LDL cholesterol, and total cholesterol observed in the elevated Lp(a) group highlight the cumulative contribution of lipid abnormalities to poor cardiovascular outcomes, corroborating previous findings [19].

Our study found that the increased Lp(a) group had higher levels of triglycerides, LDL cholesterol, and total cholesterol, consistent with earlier research that showed the cumulative impact of lipid abnormalities on poor cardiovascular outcomes [20]. This study extends prior research by reaffirming the association between elevated Lp(a) levels and adverse cardiovascular outcomes and elucidating the interplay between traditional risk factors and their exacerbating effects on Lp(a)-mediated risks. These findings underscore the importance of a comprehensive, multifaceted approach to risk stratification and management in patients with elevated Lp(a) undergoing PCI.

Although elevated Lp(a) has been identified as an independent risk factor for adverse cardiovascular outcomes, there are currently no FDA-approved therapies specifically targeting elevated Lp(a). Existing clinical guidelines recommend addressing modifiable cardiovascular risk factors in patients with high Lp(a) levels to reduce the overall risk of MACE. Management strategies include optimal control of hypertension, diabetes, and dyslipidemia, as well as encouraging smoking cessation. Statins remain a cornerstone in treating dyslipidemia, although they have a limited impact on Lp(a) levels [21]. For patients requiring additional lipid-lowering therapies, PCSK9 inhibitors have shown promise in reducing both LDL cholesterol and Lp(a) levels, though their primary indication is lowering LDL-C [22]. The emerging class of Lp(a)-lowering therapies, such as antisense oligonucleotides and siRNA-based therapies, holds the potential for directly targeting elevated Lp(a). However, these therapies are still under investigation. While lifestyle modifications play a supportive role in managing cardiovascular risk, further studies are needed to determine whether these interventions can significantly lower MACE in patients with high Lp(a). Given the limitations of current treatment options, tertiary prevention strategies, including close monitoring, regular follow-up, and prompt intervention for MACE, remain critical for patients with elevated Lp(a).

Strengths and limitations

This study provides valuable insights into the role of elevated Lp(a) levels as an independent risk factor for MACE in patients undergoing PCI. The study’s prospective design, coupled with comprehensive follow-up, strengthens the reliability of the data. We also included a well-defined cohort with precise baseline Lp(a) measurements, which allowed for more accurate analysis of the relationship between elevated Lp(a) levels and cardiovascular outcomes. Moreover, including conventional cardiovascular risk factors and their interaction with Lp(a) enhances the clinical relevance of our findings.

There are some limitations to consider. One of the main limitations is the relatively small sample size, which may underpower subgroup analyses and limit the generalizability of the findings to broader populations. Another limitation is the absence of genetic data on Lp(a), which could provide further insights into the underlying mechanisms contributing to elevated levels. Moreover, the lack of specific therapies targeting Lp(a) at the time of the study represents a gap in treatment options. While we discussed current management strategies focusing on conventional cardiovascular risk factors, the lack of FDA-approved treatments for elevated Lp(a) limits the ability to offer direct interventions for this population. However, emerging therapies may provide new options in the near future.

## Conclusions

This study highlights the substantial impact of elevated Lp(a) levels on the incidence of MACE in patients undergoing PCI. Elevated Lp(a) was associated with lower event-free survival rates and a significant increase in the risk of myocardial infarction, stroke, recurrent revascularization, and cardiovascular mortality. The detrimental effects of elevated Lp(a) were further amplified when combined with traditional cardiovascular risk factors, such as hypertension, diabetes, and dyslipidemia. These findings underscore the importance of comprehensive cardiovascular risk assessment in PCI patients, suggesting that elevated Lp(a) should be considered a key factor in clinical decision-making.

Given the long-term risks associated with elevated Lp(a), clinicians should consider periodic monitoring of Lp(a) levels in PCI patients, particularly those with additional risk factors. Intensified management strategies targeting hypertension, diabetes, and dyslipidemia could further reduce the overall cardiovascular risk in these high-risk patients. While current therapies do not directly target Lp(a), future research into Lp(a)-lowering therapies is essential. Additionally, studies should explore the role of Lp(a) in diverse populations and its interaction with other non-genetic factors. Ultimately, these findings support the need for closer follow-up and personalized treatment approaches for PCI patients with elevated Lp(a) to mitigate MACE risk and improve long-term outcomes.
